# Mechanism of Action of 2-Aminobenzamide HDAC Inhibitors in Reversing Gene Silencing in Friedreich’s Ataxia

**DOI:** 10.3389/fneur.2015.00044

**Published:** 2015-03-05

**Authors:** Elisabetta Soragni, C. James Chou, James R. Rusche, Joel M. Gottesfeld

**Affiliations:** ^1^Department of Cell and Molecular Biology, The Scripps Research Institute, La Jolla, CA, USA; ^2^Repligen Corporation, Waltham, MA, USA

**Keywords:** Friedreich’s ataxia, histone deacetylase inhibitor, epigenetics, 2-aminobenzamide, frataxin

## Abstract

The genetic defect in Friedreich’s ataxia (FRDA) is the hyperexpansion of a GAA•TTC triplet in the first intron of the *FXN* gene, encoding the essential mitochondrial protein frataxin. Histone post-translational modifications near the expanded repeats are consistent with heterochromatin formation and consequent *FXN* gene silencing. Using a newly developed human neuronal cell model, derived from patient-induced pluripotent stem cells, we find that 2-aminobenzamide histone deacetylase (HDAC) inhibitors increase *FXN* mRNA levels and frataxin protein in FRDA neuronal cells. However, only compounds targeting the class I HDACs 1 and 3 are active in increasing *FXN* mRNA in these cells. Structural analogs of the active HDAC inhibitors that selectively target either HDAC1 or HDAC3 do not show similar increases in *FXN* mRNA levels. To understand the mechanism of action of these compounds, we probed the kinetic properties of the active and inactive inhibitors, and found that only compounds that target HDACs 1 and 3 exhibited a slow-on/slow-off mechanism of action for the HDAC enzymes. HDAC1- and HDAC3-selective compounds did not show this activity. Using siRNA methods in the FRDA neuronal cells, we show increases in *FXN* mRNA upon silencing of either HDACs 1 or 3, suggesting the possibility that inhibition of each of these class I HDACs is necessary for activation of *FXN* mRNA synthesis, as there appears to be redundancy in the silencing mechanism caused by the GAA•TTC repeats. Moreover, inhibitors must have a long residence time on their target enzymes for this activity. By interrogating microarray data from neuronal cells treated with inhibitors of different specificity, we selected two genes encoding histone macroH2A (*H2AFY2*) and Polycomb group ring finger 2 (*PCGF2*) that were specifically down-regulated by the inhibitors targeting HDACs1 and 3 versus the more selective inhibitors for further investigation. Both genes are involved in transcriptional repression and we speculate their involvement in *FXN* gene silencing. Our results shed light on the mechanism whereby HDAC inhibitors increase *FXN* mRNA levels in FRDA neuronal cells.

## Introduction

Friedreich’s ataxia (FRDA, OMIM 229300) is an autosomal recessive neurodegenerative disorder caused by a GAA•TTC triplet repeat expansion in an intron of the nuclear *FXN* gene, which encodes the essential mitochondrial protein frataxin (1). Frataxin is involved in the assembly of iron–sulfur clusters, and their transfer to mitochondrial enzymes and components of the electron transport chain [reviewed in Ref. (2)]. Unaffected individuals have between 6 and 30 GAA•TTC repeats, whereas affected individuals have from approximately 70 to more than 1,000 triplets. A small number of patients are compound heterozygous, with one expanded *FXN* allele and an inactivating point mutation on the second allele. The effect of the GAA•TTC expansion mutation is to reduce expression of frataxin at the level of transcription (3), through the formation of heterochromatin and subsequent gene silencing (4–8). Frataxin insufficiency leads to decreased activity of iron–sulfur cluster enzymes, mitochondrial iron accumulation, and resultant cell death, with the primary sites of pathology being the large sensory neurons of the dorsal root ganglia and the dentate nucleus of the cerebellum (9). Non-neuronal tissues are also involved in the disease. Cardiomyopathy is common among FRDA patients and diabetes is found in 10% of FRDA patients (10, 11). Approximately, 60% of patients succumb to the disease in early adulthood due to cardiomyopathy (12). Currently, there is no approved and effective therapy for this disorder.

The epigenetic basis for transcriptional silencing in FRDA is now well established (5–8, 13, 14). The GAA•TTC repeat expansion is correlated with both increased DNA methylation in the region of *FXN* intron 1 immediately upstream of the GAA•TTC repeats (6, 7, 15), as well as with reduced histone acetylation and increased histone trimethylation at the *FXN* promoter (6, 8), and in intron 1 adjacent to the repeats (5–7). A number of hypotheses have been put forward to explain how the GAA•TTC repeats induce heterochromatin formation, but this remains an open question. Attractive hypotheses for induction of silencing include RNA-mediated silencing, where either sense or antisense transcription of the repeats initiates an RNA-induced silencing complex (8). Alternatively, the repeats could form non-B DNA structures, which induce silencing. An extensive literature documents the formation of triplex or “sticky DNA” structures by GAA•TTC repeat DNA (16). Lastly, recent evidence suggests that R-loops can mediate heterochromatin formation and gene silencing (17).

Based on this large body of evidence for the mechanism of gene silencing in FRDA [reviewed in Ref. (13, 18, 19)], it was reasonable to propose epigenetic-modifying compounds as a potential therapeutic strategy for FRDA. In an early study, Sarsero and colleagues (20) tested sodium butyrate for its ability to increase *FXN* mRNA expression, but only a modest effect was observed. Our laboratory reported a screen of a panel of commercially available histone deacetylase (HDAC) inhibitors in FRDA lymphoblasts and we found that only the benzamide BML-210 [*N*^1^-(2-aminophenyl)-*N*^8^-phenyloctanediamide] produced a significant increase of *FXN* mRNA expression in FRDA lymphoblasts (5). Similarly, Festenstein and co-workers have reported that the sirtuin protein deacetylase inhibitor nicotinamide (vitamin B3) also increases *FXN* mRNA levels in FRDA lymphoblasts, in a FRDA mouse model (14), and recently in circulating lymphocytes from nicotinamide-treated patients (21).

Our laboratory identified a 2-aminobenzamide HDAC inhibitor, *4b* [*N*^1^-(2-aminophenyl)-*N*^7^-phenylheptanediamide], which was shown to act on FRDA primary lymphocytes to significantly increase acetylation of H3K14, H4K5, and H4K12 in the *FXN* upstream GAA•TTC region and to significantly increase *FXN* mRNA levels (5). Further development of this family of 2-aminobenzamide HDAC inhibitors identified other compounds, which have shown efficacy in FRDA patient cells and in mouse models (22–27). These compounds produce significant short-term increases in histone acetylation and *FXN* mRNA and frataxin protein expression in FRDA primary lymphocytes and brain and heart tissues of FRDA KIKI mice (23, 26), and have also shown efficacy in a transgenic mouse model for FRDA (27). More recently, we showed efficacy of a 2-aminobenzamide HDAC inhibitor (HDACi *109*) in neuronal cells derived from FRDA-induced pluripotent stem cells (iPSCs) (28). This compound was taken into a Phase Ib clinical trial in FRDA patients, where drug treatment lead to increases in *FXN* mRNA and histone acetylation at the *FXN* gene in peripheral blood mononuclear cells. Interestingly, the concentration of drug required to induce epigenetic changes in neuronal cells is comparable to the exposure in patients required to observe increases in histone acetylation and gene activation. While the 2-aminobenzamides are promising therapeutics for FRDA, further development of this compound class will be necessary to identify molecules for chronic use. Here, we explore the mechanism of action of this compound class and our efforts to identify improved molecules for future clinical study.

## Materials and Methods

### Materials

Recombinant human HDAC1 and HDAC3/NCoR2, expressed in baculovirus, were purchased from BPS Bioscience (San Diego, CA, USA). The HDAC inhibitors *109*, *136*, *3*, *233*, *966*, and *Click-1* were synthesized as previously described (25, 26, 29–31).

### HDAC activity assays

The deacetylase activities of HDACs 1 and 3 were measured by assaying enzyme activity using Lys-C peptidase and the synthetic substrate acetyl-Lys(Ac)-AMC, as previously described (22). The HDAC enzymes produce deacetylated lysine-AMC, which can be cleaved by the peptidase to generate free fluorogenic 4-methylcoumarin-7-amide (MCA). MCA fluorescence is read with an excitation wavelength of 370 nm and emission wavelength of 460 nm, using a Tecan M200 96 well-plate reader (San Jose, CA, USA). All HDAC assays were performed in 96 well, non-binding plates (Greiner Bio-one, NC, USA) in 50 mM Tris–HCl buffer (pH 8.0), containing 137 mM NaCl, 1 mM MgCl_2_, 2.7 mM KCl, and 0.1 mg mL^−1^ bovine serum albumin at ambient temperature. To determine inhibition mechanisms and associated kinetic values, a series of enzyme progression curves for HDACs 1 and 3, at different concentrations of inhibitors, were generated by adding 100 ng of each enzyme into separate reaction mixtures containing 50 μM acetyl-Lys(Ac)-AMC substrate (five times the *K*_m_) and 2 mU of Lys-C peptidase developer for a period of 1 h. Data from each progression curve, at different inhibitor concentrations, were fit using the non-linear regression program KaleidaGraph to the integrated rate equation for slow-binding inhibitors:

(1) $$\left[F\right]=v_{s}t+\left(v_{0}-v_{s}\right)\left(1-\exp\;\left(-k_{\mathrm{obs}}t\right)\right)∕k_{\mathrm{obs}}$$

where [*F*] is the amount of MCA fluorophore generated, represented in arbitrary fluorescence units (AFU, *r*), which is proportion to the deacetylated substrate at time *t*. *v*_0_ and *v*_s_ are the initial and the final steady-state velocities, respectively. *k*_obs_ is the apparent first-order rate constant obtained by the best fit to the data. The *k*_obs_ values were then plotted against the inhibitor concentrations for which each *k*_obs_ value was obtained. For mechanism 1 (see below), the relationship between *k*_obs_ and the inhibitor concentration is linear:

(2) $$k_{\mathrm{obs}}=k_{-1}+k_{1}\left[I\right]∕\left(1+\left[S\right]∕K_{m}\right)$$

and

(3) $$K_{i}=k_{-1}∕k_{1}$$

For mechanism 2, the relationship between *k*_obs_ and the inhibitor concentration is hyperbolic:

(4) $$k_{\mathrm{obs}}=k_{-2}+k_{2}\left[I\right]∕\left[\left[I\right]+K_{\text{i}}^{*}\left(1+\left[S\right]∕K_{m}\right)\right]$$

and

(5) $$K_{i}={K_{i}}^{*}\left[k_{-2}∕\left(k_{2}+k_{-2}\right)\right]$$

where $K_{\text{i}}^{*}$ is the stable complex forming constant and *K*_i_ is the overall final inhibitory constant for the entire process.

### Derivation of FRDA neuronal cells, qRT-PCR, and western blotting

All methods for iPSC derivation (32) and neuronal differentiation (28, 33) have been presented. Two patient fibroblast lines (GM03816 and GM04878 from the Coriell repository) and one unaffected fibroblast line (GM08333 from the Coriell repository) were used to derive iPSCs and neurons (as approved by the University of California, San Diego, Human Research Protection Program, Embryonic Stem Cell Research Oversight Committee, project #110235ZO). Quantitative RT-PCR was performed as described (28), with the following primer pairs:

*FXN*: 5′-CAGAGGAAACGCTGGACTCT-3′ and 5′-AGCCAGATTTGCTTGTTTGG-3′
*HDAC1*: 5′-CCGCATGACTCATAATTTGC-3′ and 5′-GGTCATCTCCTCAGCATTGG-3′
*HDAC2*: 5′-CGCATGACCCATAACTTGC-3′ and 5′-TGTCATTTCTTCGGCAGTGG-3′
*HDAC3*: 5′-GTATGAAGTCGGGGCAGAGA-3′ and 5′-GGCTGGAAAAGGTGCTTGTA-3′
*PCGF2*: 5′-AGCATCAGGTCTGACAAAACAC-3′ and 5′-GCCGCCGTTTCATCTCATC-3′
*H2AFY2*: 5′-GCGGCAGTCATTGAGTACCTG-3′ and 5′-CAAGATGTGTCTCGGGGCTAT-3′

Data were normalized to RNA concentration and *p* values calculated using the *t*-test. Western blotting was performed as described (28), with antibodies to HDACs 1, 2, and 3 from Abcam (ab7028, ab7029, ab7030, respectively), used at a dilution of 1:5000. RNA polymerase II signal was used as a recovery standard and antibodies against RNA polymerase II were purchased from Millipore (05-952) and used at a dilution of 1:2000. Protein concentrations were determined with the BCA assay (Thermo Scientific).

### siRNA-mediated down-regulation of HDACs 1, 2, and 3

siRNAs were purchased from Life Technologies (HDAC1 siRNA ID # 120418, HDAC2 siRNA ID # 120208, HDAC3 siRNA ID #120349). Neuronal cells were allowed to differentiate for 10 days in Neurobasal A medium supplemented with N2 and B27 (all from Life Technologies) before addition of siRNAs to a final concentration of 12 nM and lipofectamine RNAiMAX transfection reagent (Life Technologies). The next day media was changed and cells were allowed to recover for another 24 h. At day 12 of differentiation, another round of siRNA transfection was performed as above. Cells were collected for qRT-PCR and western blotting analyses 96 h after the first siRNA treatment.

### Chromatin immunoprecipitation

Chromatin immunoprecipitation (ChIP) is described in detail in Soragni et al. (28). The antibodies used in this study are the same as the ones indicated in the western blotting section, each used at a concentration of 5 μg mL^−1^. Samples were quantified in triplicate by real-time PCR using the standard curve method. The DNA recovery for each region is expressed as the ratio to the DNA recovery of GAPDH coding region. The primers used were:

*FXN* promoter: 5′-CCCCACATACCCAACTGCTG-3′ and 5′-GCCCGCCGCTTCTAAAATTC-3′
UPGAA: 5′-GAAACCCAAAGAATGGCTGTG-3′ and 5′-TTCCCTCCTCGTGAAACACC-3′
DOWNGAA: 5′-CTGGAAAAATAGGCAAGTGTGG-3′ and 5′-CAGGGGTGGAAGCCCAATAC-3′
p21(CDKN1A): 5′-GCGTTCACAGGTGTTTCTGC-3′ and 5′-ACATCCCGACTCTCGTCACC-3′
MYC promoter: 5′-TGCGATGATTTATACTCACAGG-3′ and 5′-CTCCCTCTCAAACCCTCTCC-3′
GAPDH: 5′-CACCGTCAAGGCTGAGAACG-3′ and 5′-ATACCCAAGGGAGCCACACC-3′

### Microarray analysis

Eight-day-old neurons were treated with DMSO or 10 μM *109*, *966*, or *233* for 24 h. RNA purification, labeling, and hybridization to Illumina HT-12 v4 arrays (see: http://www.illumina.com/products/humanht_12_expression_beadchip_kits_v4.html) were performed as previously described (32). The HT-12 array targets more than 31,000 annotated genes and splice variants, with more than 47,000 probes [derived from the National Center for Biotechnology Information (NCBI) Reference Sequence (RefSeq) Release 38, November 7, 2009]. After normalization, genes differentially expressed between the DMSO groups (three replicates) and the *109*-treated groups (three replicates) were identified with a Student’s *t*-test at a significance level of *p* < 0.01 (uncorrected *p* value). Genes with expression level changes greater than 2 in the *109* groups and smaller that 1.5 in the *233* or *966* groups (one replicate per compound) were then subjected to functional annotation analysis via the Database for Annotation, Visualization, and Integrated Discovery (DAVID). Illumina probes were converted to gene symbols using DAVID gene ID conversion tool. Microarray data are deposited in the Gene Expression Omnibus as accession number GSE65399.

## Results

### Isotype selectivity for HDAC inhibitors that increase *FXN* gene expression in FRDA cells

**Figure 1:**
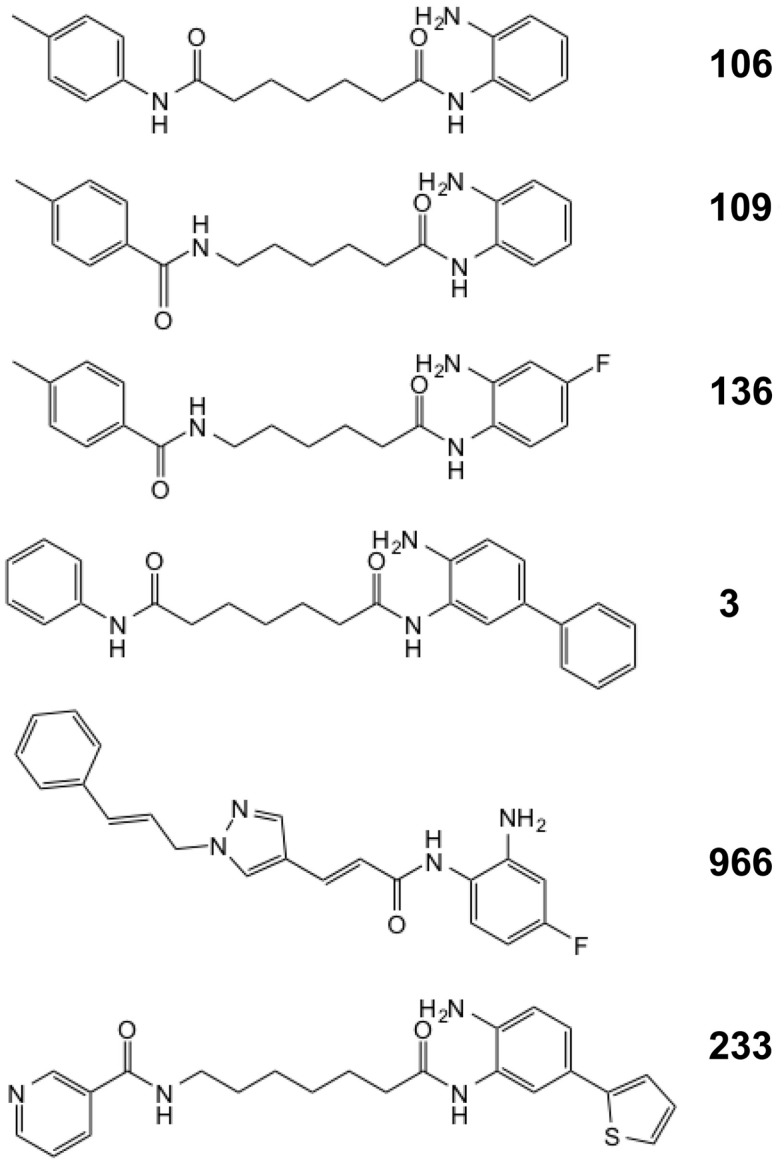
**Structures of the HDAC inhibitors**.

We previously described a series of 2-aminobenzamide HDAC inhibitors that have differential inhibitory activities against members of the class I HDAC enzymes (25, 26). We synthesized a series of focused chemical libraries to provide diversity in potency, selectivity, and pharmacologic properties with the aim of developing a pharmaceutical agent for the treatment of FRDA. The general structure exemplified by HDACi *106* (Figure 1) shows the salient features of the pimelic 2-aminobenzamide family: the solvent accessible or cap group (left ring), linker group (five methylenes), and zinc-binding group (2-aminophenyl). By analogy to the hydroxamate HDAC inhibitors for which co-crystal structural information has been obtained (34, 35), and the recent crystal structure of HDAC3 (36), the right side of the molecule interacts with the Zn-containing active site of HDAC enzymes while the left side is positioned to interact with protein surface residues distant from the active site. Substitution at the 4-position of the 2-aminobenzamide ring with fluorine increases selectivity for HDAC3 inhibition by decreasing potency to inhibit HDAC1/2 (26, 37). Large substitutions at the 5-position, such as a phenyl or thiophene increase selectivity for HDAC1/2 over HDAC3 (25, 29).

In recent studies, we found that only compounds that target each of the class I HDACs (HDACs 1, 2, and 3) are potent activators of *FXN* gene expression in iPSC-derived FRDA neuronal cells (28). To further explore the relationship between HDAC isotype selectivity and *FXN* gene expression, we focus on three compounds, *109* [*N*-(6-(2-aminophenylamino)-6-oxohexyl)-4-methylbenzamide; (26, 28)], *136* [*N*-(6-(2-amino-4-fluorophenylamino)-6-oxohexyl)-4-methylbenzamide; (26)], and *3* [*N*^1^-(4-aminobiphenyl-3-yl)-*N*^7^-phenylheptanediamide; (25)]. Compound structures are shown in Figure 1 and IC_50_ and *K*_i_ values for inhibition of HDACs 1 and 3 are shown in Table 1. While HDACi *109* is a potent inhibitor of both enzymes, compound *136* is much less potent than *109* with a slight selectivity for HDAC3 and *3* is a selective inhibitor for HDAC1 (25, 26).

**Table 1:** **Inhibition constants, IC_50_, and *K*_i_ values for HDAC inhibitors and HDACs 1 and 3**.

Compound	IC_50_ HDAC1	IC_50_ HDAC3	Selectivity HDAC3	*K*_i_ HDAC1	*K*_i_ HDAC3	Selectivity HDAC3
109^a^	60 nM	50 nM	~1	32 nM	5 nM	~6
136^a^	1.14 μM	560 nM	~2	630 nM	196 nM	~3
3^b^	127 nM	9.6 μM	0.013	7 nM	2.5 μM	0.028

*^a^Data from Rai et al. (26)*.

*^b^Data from Xu et al. (25)*.

**Figure 2:**
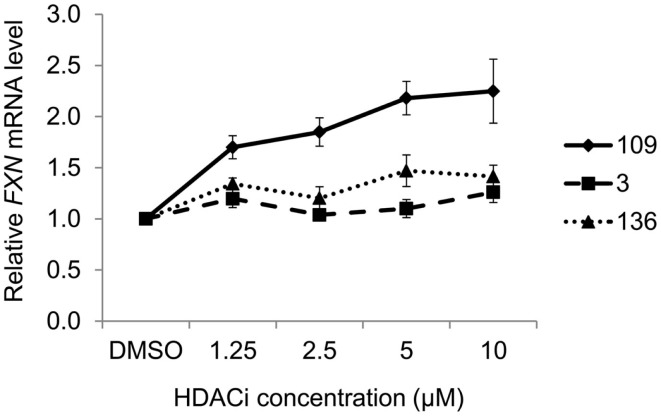
**Effect of HDAC inhibitors on *FXN* mRNA levels in neuronal cells**. Quantitative RT-PCR analysis of *FXN* expression in 16-day-old FRDA neurons after 24-h treatment with HDAC inhibitors *109*, *136*, and *3* at varying concentrations. *FXN* mRNA levels were normalized to total RNA concentration. Signals from DMSO-treated samples were arbitrarily set to 1. Error bars = SEM of triplicate measurements.

Figure 2 presents the results of qRT-PCR analysis of *FXN* mRNA levels after incubation of FRDA iPSC-derived neurons with each of the three HDACi for 24 h. As expected from previous studies with other isotype selective compounds (28), only HDACi *109* is a potent inducer of *FXN* gene expression in these cells, while compounds *136* and *3* had either no effect (*3*) or only a modest effect (*136*) on *FXN* mRNA levels. None of these compounds have pronounced effects on *FXN* gene expression in cells from unaffected individuals (25, 26, 28). These findings are also similar to our results with HDACi *966* [(*E*)-*N*-(2-amino-4-fluorophenyl)-3-(1-cinnamyl-1*H*-pyrazol-4-yl)acrylamide; ~30-fold selectivity for HDAC3 over HDAC1/2) (37)] and *233* [*N*-(2-amino-5-(2-thienyl)phenyl)-7-nicotinoylamino-heptanamide; >200-fold selectivity for HDAC1 over HDAC3 (30)]. We conclude that simultaneous inhibition of HDACs 1 and 3 is necessary for reactivation of *FXN* gene expression in the neuronal cell model.

In a previous study (22), we analyzed the mechanism of enzyme inhibition for HDACi *106* for both HDAC1 and HDAC3-NCoR. Compounds *106* and *109* are constitutional isomers of each other, where the orientation of the “left” amide linkage is reversed in the two molecules (Figure 1). We previously reported that compound *106* is a slow-on/slow-off inhibitor (22). There are two common mechanisms for slow-on/tight-binding inhibitors, as shown below. Mechanism 2 differs from mechanism 1 in that a stable enzyme-inhibitor complex (EI*) is formed, whereas no such stable intermediate is formed in mechanism 1 (38). The equations describing these mechanisms, and the derivation of inhibition constants, on-rates, and off-rates from progression curve HDAC inhibition assays, are described above. Briefly, for mechanism 1, the relationship between *k*_obs_ and the inhibitor concentration is linear, whereas for mechanism 2, the relationship between *k*_obs_ and inhibitor concentration is hyperbolic. In contrast to these mechanisms, a fast-on/fast-off competitive inhibition mechanism is best described by a plot of *v*_i_/*v*_0_ against inhibitor concentration, where *v*_i_ and *v*_0_ are the reaction velocities (deacetylation rates) in the presence or absence of the inhibitor, respectively.

#### Mechanism 1


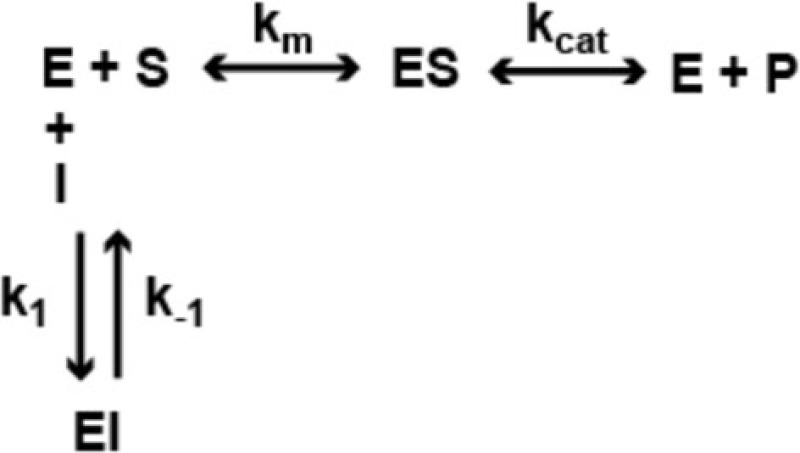


#### Mechanism 2


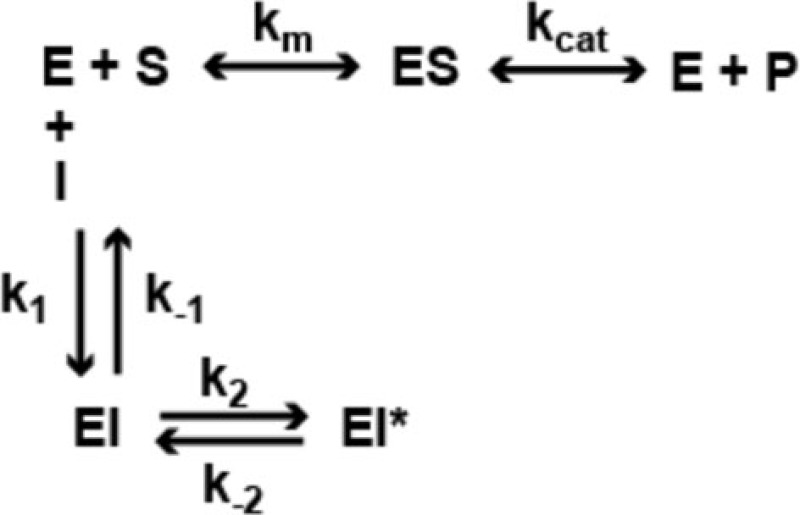


IC_50_ determinations showed time-dependence for inhibition for HDAC1 *109* with both HDAC1 and HDAC3-NCoR, indicating a slow-on mechanism (26). From kinetic measurements, we showed that *109* is a slow-on/slow-off inhibitor of both enzymes, but the on-rate of *109* is faster for HDAC1 than for HDAC3/NCoR. Compound *109* has a *K*_i_ of 32 nM for HDAC1 and a *K*_i_ of 5 nM for HDAC3/NCoR2, resulting in an approximately 6-fold selectivity for HDAC3 over HDAC1 (Table 1). Kinetic constants for *109* are provided in Table 2. Plots of *k*_obs_ versus inhibitor concentration are provided in Figures S1–S3 in Supplementary Material for HDACi *109*, *136*, and *3*, respectively. Plots of *k*_obs_ versus inhibitor concentration for HDACi *109* with either HDAC1 or HDAC3-NCoR are best fit by the equation for mechanism 2, indicating a stable EI* complex is formed with both enzymes. In other words, *109* is a slow-on/tight-binding inhibitor of both enzymes.

**Table 2:** **Inhibition constants for benzamide HDAC inhibitors**.

Compound	HDAC3	HDAC1
**109**	*k*_−2_ = 0.0025 min^−1^	*k*_−2_ = 0.0036 min^−1^
	*k*_2_ = 0.0762 min^−1^	*k*_2_ = 0.121 min^−1^
	mechanism 2	mechanism 2
**136**	*k*_−2_ = 0.0136 min^−1^	Fast-on/fast-off
	*k*_2_ = 0.124 min^−1^	Competitive inhibition
	mechanism 2	mechanism
**3**	*k*_−1_ = 0.0015 min^−1^	*k*_−2_ = 0.0017 min^−1^
	*k*_1_ = 600 min^-1 ^M^−1^	*k*_2_ = 0.1675 min^−1^
	mechanism 1	mechanism 2

In IC_50_ determinations, compound *136* shows a time-dependent inhibition of HDAC3/NCoR, where the IC_50_ against HDAC3/NCoR decreases from ~17 μM to 560 nM over a period of 3 h; however, no time-dependent inhibition of HDAC1 was observed with compound *136* (26). Kinetic measurements (Table 2; Figure S2 in Supplementary Material) showed that compound *136* is a fast-on/fast-off, competitive inhibitor of HDAC1 with *K*_i_ of 630 nM, whereas *136* is a slow-on/slow-off, tight-binding inhibitor of HDAC3/NCoR with *K*_i_ of 196 nM, resulting in a ~3-fold selectivity for HDAC3 over HDAC1, following inhibition mechanism 2 only for HDAC3 (Figure S2 in Supplementary Material). The competitive inhibition mechanism for *136* with HDAC1 is similar to the inhibition mechanism for SAHA and other hydroxamate HDAC inhibitors that fail to activate *FXN* gene expression (5, 22). Appending a phenyl group at the 5-position of the benzamide ring of HDACi *106* (22) resulted in compound *3*, with increased selectivity for HDAC1/2 over HDAC3 (25). HDACi *3* has a ~75-fold preference for HDAC1 over HDAC3 comparing IC_50_ values and a ~350-fold preference for HDAC1 over HDAC3 from *K*_i_ measurements (Table 1). Similar to *109* and *136*, the kinetic data for *3* and HDAC1 (Table 2) are best fit to a slow-on/slow-off inhibition mechanism 2, whereas the kinetic data showed that HDACi *3* inhibits HDAC3 via mechanism 1, where no stable complex is formed (Figure S3 in Supplementary Material). We thus conclude that compounds that are potent activators of *FXN* gene expression in FRDA cell models share a common property of inhibition of class I HDACs through inhibition mechanism 2 and compounds that are inactive in *FXN* gene expression assays fail to inhibit either HDAC1 or HDAC3 by this mechanism. This suggests that stable target engagement is a requirement for activation of *FXN* gene expression and that inhibition of both HDAC1 and HDAC3 is required for *FXN* gene reactivation.

### Effects of siRNA gene silencing on *FXN* mRNA

**Figure 3:**
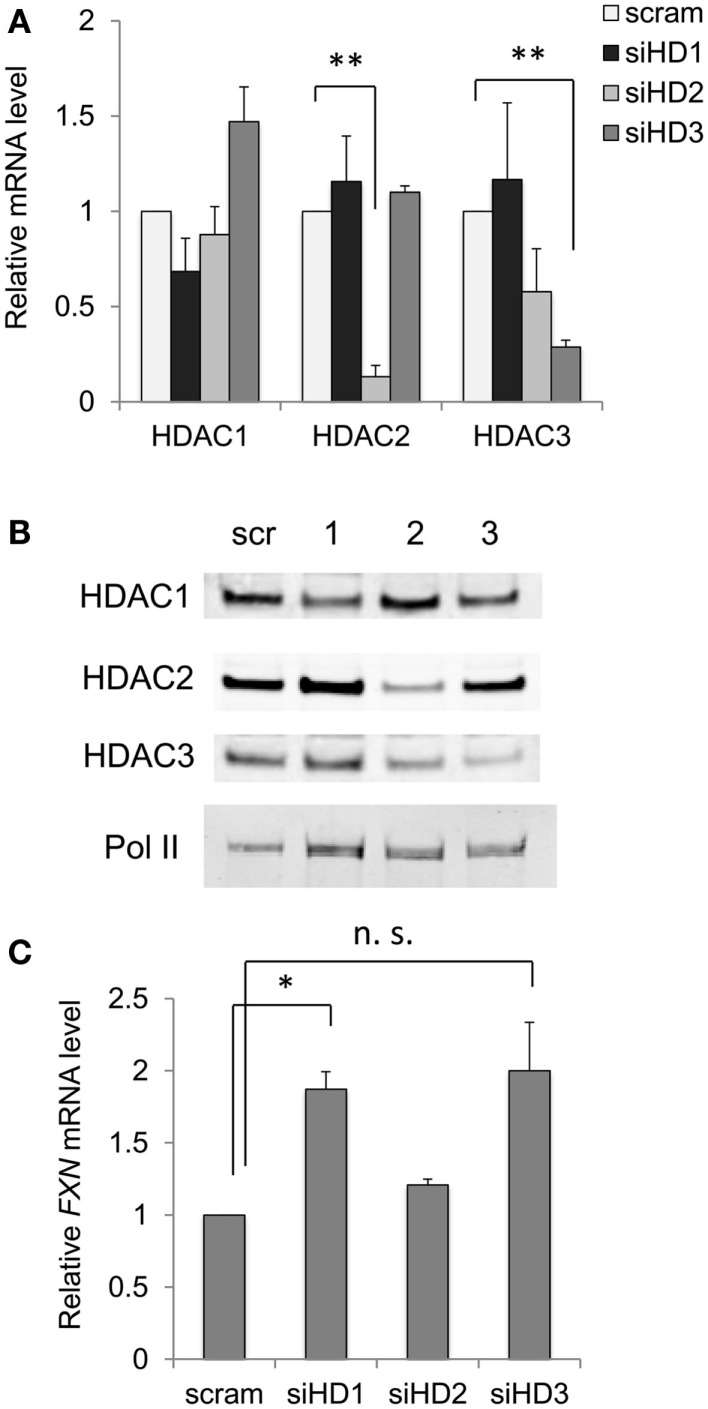
**Effects of down-regulation of HDAC enzyme levels by siRNA on *FXN* mRNA**. **(A)** Effects of siRNAs to HDACs 1–3 on the mRNA levels for each HDAC enzyme in FRDA neuronal cells. Quantitative RT-PCR was used to determine mRNA levels, with each mRNA arbitrarily set to 1 in the presence of a scrambled siRNA (scram). Error bars = SEM of duplicate biological experiments, quantified in triplicate. **p < 0.01. **(B)** Western blots for HDAC1, HDAC2, HDAC3, and RNA polymerase II (protein recovery standard), after treatment with a scrambled (Scr) siRNA, or siRNAs to HDACs 1–3 as indicated at the top of the figure. One example is shown, but the experiment was performed twice. **(C)** *FXN* mRNA levels were determined in FRDA neuronal cells by qRT-PCR after treatment with scrambled (scram) or siRNAs to HDAC 1–3, as indicated (siHD1-3). Error bars = SEM of duplicate biological experiments, quantified by qRT-PCR in triplicate. *p < 0.05. n.s., not significant, see text.

If this latter assertion is correct, then down-regulation of class I HDAC enzyme levels in neuronal cells by siRNA methods should yield a similar result as obtained with small-molecule inhibitors. Figure 3 shows the effect of siRNA targeting each of the class I HDACs with validated siRNAs, at both the mRNA level (Figure 3A) and protein levels (Figure 3B). While siHDAC2 and siHDAC3 reduce the mRNAs for their cognate enzymes by greater than 70% compared to a control, scrambled siRNA, siHDAC1 was less active, achieving only 30% knockdown. Curiously, siHDAC2 also caused a non-significant reduction in HDAC3 mRNA levels (also seen at the protein level, Figure 3B), but not to the extent of HDAC3 knockdown. We next monitored the effects of these siRNAs at the level of their cognate proteins by western blotting with well-established antibodies [see Materials and Methods and Ref. (28)]. Each siRNA had significant effects on enzyme levels, with clear specificity for the targeted enzyme. Only siHDAC2 appeared to also down-regulate HDAC3 as well as HDAC2, as found for mRNA levels. With these results in hand, we monitored the levels of *FXN* mRNA in knockdown neuronal cells. We find that siHDAC1 and siHDAC3 were potent inducers of *FXN* gene expression (Figure 3C), although *FXN* up-regulation with siHDAC3 treatment did not reach statistical significance (*p* value = 0.09). In contrast, siHDAC2 did not increase *FXN* mRNA to the extent observed with the other siRNAs. Previous studies in various systems have pointed to differences between small-molecule inhibition and protein ablation in terms of biological outcome (39). Removing a particular HDAC enzyme from the cell will naturally affect its interaction partners, many of which are other HDACs or components of shared co-repressor complexes (40). Thus, it is not surprising that down-regulation of either HDAC1 or HDAC3 can result in potent increases in *FXN* gene expression in FRDA cells, pointing again to the involvement of the class I HDACs in GAA•TTC repeat-mediated *FXN* gene repression.

### Localization of HDAC enzymes on the FXN gene

**Figure 4:**
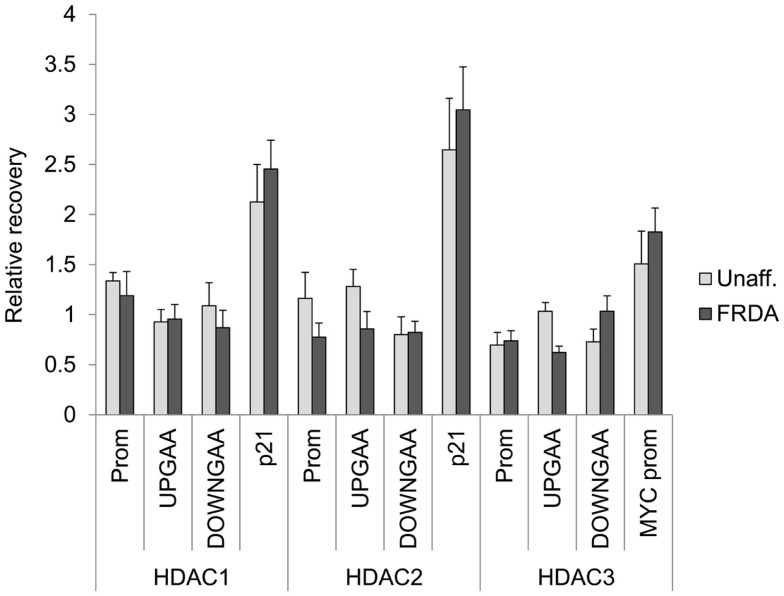
**Class I HDACs are not detected on the *FXN* gene in neuronal cells**. ChIP was performed on 16-day-old neurons with antibodies against HDAC1, HADC2, and HDAC3. The region of the promoter (Prom), upstream (UPGAA), and downstream (DOWNGAA) of the GAA•TCC repeats on the *FXN* gene were interrogated by qPCR. Data are plotted as enrichment over the DNA recovery of the *GAPDH* coding region. The recovery of *CDKNA1* (p21) promoter and *MYC* promoter are shown as positive controls.

To ask whether the class I HDACs interact directly with the *FXN* gene in FRDA neuronal cells, we performed ChIP studies. Our previous ChIP studies clearly showed increases in histone acetylation along the *FXN* gene after incubation with HDAC inhibitors that increase *FXN* gene expression (5, 28). We also found that hydroxamates such as SAHA and TSA that are without effect on *FXN* gene expression also failed to increase acetylation of histones H3 and H4 at the *FXN* gene locus in lymphoid cells (5), and isotype-selective HDACi allowed us to identify lysines 9 and 27 of histone H3 as critical residues for *FXN* reactivation (28). In contrast to these clear results, ChIP studies consistently failed to provide evidence for localization of class I HDACs either upstream or downstream of the GAA•TTC repeats (Figure 4). Since these repeats are ubiquitous in the human genome, it is not possible to assess HDAC enzyme occupancy at the *FXN* repeats by ChIP, so it is conceivable that these enzymes do localize with the repeats. Nonetheless, in these experiments, we attempted to shear chromatin fragments to lengths that would include the repeats when probing with primer sets either upstream or downstream from the repeats (28, 32). These findings are consistent with a transitory interaction between class I HDACs and *FXN* gene chromatin or perhaps indicate an indirect mechanism of action. The increase in histone acetylation that we see upon HDACi treatment (5, 28) can be a direct effect of inhibition of HDAC complexes residing on the *FXN* gene, or a consequence of gene reactivation via a different mechanism.

### Do HDAC inhibitors act directly on the *FXN* gene or through different genes?

**Figure 5:**
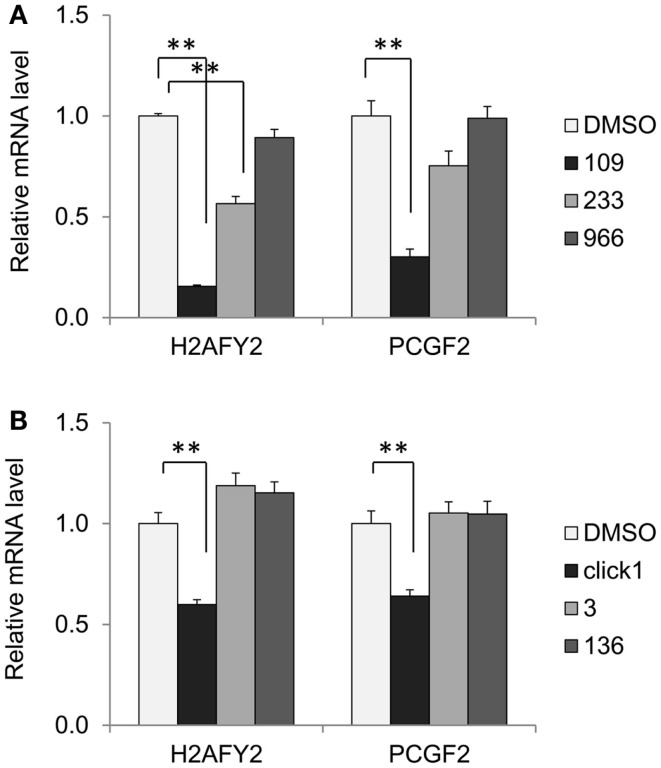
**HDACi *109* down-regulates genes involved in chromatin structure**. **(A)** Effect of HDAC inhibitors *109*, *233*, and *966* on mRNA levels for H2AFY2 (histone H2A family Y isotype 2, encoding macroH2A) and PCGF2 (Polycomb group ring finger 2) in FRDA neuronal cells. Cells were treated with the indicated compounds at 10 μM for 24 h; RNA levels were quantified by qRT-PCR and mRNA levels in the DMSO control was set to 1. Error bars = SEM of triplicate measurements. **p < 0.01. **(B)** Effect of HDAC inhibitors *Click-1*, *3*, and *136* on mRNA levels for H2AFY2 and PCGF2 in FRDA neuronal cells, as in **(A)**, except the HDACi concentration was 5 μM. **p < 0.01.

While increased histone acetylation of *FXN* chromatin on treatment with our HDAC inhibitors suggests that *FXN* gene chromatin is the direct target of these compounds, an alternative hypothesis for a mechanism of action of our HDAC inhibitors is that the active molecules cause a change in expression of a gene or set of genes that in turn regulate *FXN* gene expression. To assess this possibility, we treated FRDA neuronal cells with compounds *109*, *233*, and *966* (10 μM for 24 h) and then analyzed global gene expression with Illumina microarrays. We established by qRT-PCR that HDACi *109* did indeed up-regulate *FXN* mRNA while the other two compounds did not [data not shown, but see Figure 2 and Ref. (28)]. We interrogated the microarray data first for genes that are specifically up or down-regulated by HDACi *109*, with a *p* value <0.01, and assembled a list of 1,216 such genes (661 genes are up-regulated and 555 genes are down-regulated; Table S1 in Supplementary Material). Functional annotation clustering of these genes using the DAVID, identified groups related to transcription and nuclear processes, apoptosis, and mitochondrial function (not shown). We then selected only genes that were up-regulated or down-regulated by at least 2-fold and subtracted from this list genes whose expression increased or decreased more than 1.5-fold, upon treatment with HDACi *233* or HDACi *966*. This resulted in a final list of 88 genes of which 51 were uniquely up- and 37 uniquely down-regulated by *109* (Table S2 in Supplementary Material). We then interrogated this final list for genes that might be involved in chromatin structure or gene expression using DAVID as above. Table 3 provides a list of 12 such genes, identified by functional annotation clustering, although non-significantly enriched in the 88 gene list. Two of these genes appeared to be notable to us, namely histone H2A family member Y2 (*H2AFY2*) and Polycomb group ring finger 2 (*PCGF2*). Each of these genes is down-regulated selectively by HDACi *109* but not by the other HDACi that fail to up-regulate *FXN* mRNA. *H2AFY2* encodes a macroH2A protein that has been implicated in X chromosome inactivation, gene repression, and genomic imprinting [reviewed in Ref. (41)]. PCGF2 is a negative regulator of developmentally important genes, and specifically involved in H3K27 methylation-mediated gene repression [reviewed in Ref. (42)]. To validate the microarray data, we performed qRT-PCR for *H2AFY2* and *PCGF2* mRNAs with RNA from neuronal cells that were treated with each of the three HDAC inhibitors used in the microarray study. Figure 5A shows that only compound *109* caused a significant decrease in expression of both *H2AFY2* and *PCGF2*. We then asked if these results could be reproduced with a second series of 4- and 5-substituted 2-aminobenzamides, namely, HDACi *136* and *3*. Indeed, only an active compound similar to *109* (*Click-1*, see below) was able to significantly down-regulate *H2AFY2* and *PCGF2* gene expression (Figure 5B), as predicted from the microarray data and results with HDACi *966* and *233*. While HDACi *136* was moderately active at 10 μM concentration (not shown), it was inactive at lower concentrations and not as active as HDACi *109* and *Click-1* in down-regulating expression of these two genes. Future studies will need to address whether these two proteins, macroH2A and Polycomb group ring finger 2 are indeed directly involved in *FXN* gene expression in FRDA neuronal cells. However, the present data provide a correlation between HDACi *109* treatment, up-regulation of *FXN*, and down-regulation of these two gene products.

**Table 3:** **List of genes uniquely regulated by *109* and involved in gene expression or chromatin structure**.

Gene symbol	Gene name
H2AFY2	H2A histone family, member Y2
PCGF2	Polycomb group ring finger 2
PLAGL1	Pleomorphic adenoma gene-like 1
MLXIPL	MLX-interacting protein-like
ZNF586	Zinc finger protein 586
ABT1	Activator of basal transcription 1
ZFX	Zinc finger protein, X-linked
PRKDC	Similar to protein kinase, DNA-activated, catalytic polypeptide
POU4F2	POU class 4 homeobox 2
POLE	Polymerase (DNA directed), epsilon
ZNF92	Zinc finger protein 92
TRIM25	Tripartite motif-containing 25

### Development of improved HDAC inhibitors for clinical use

**Figure 6:**
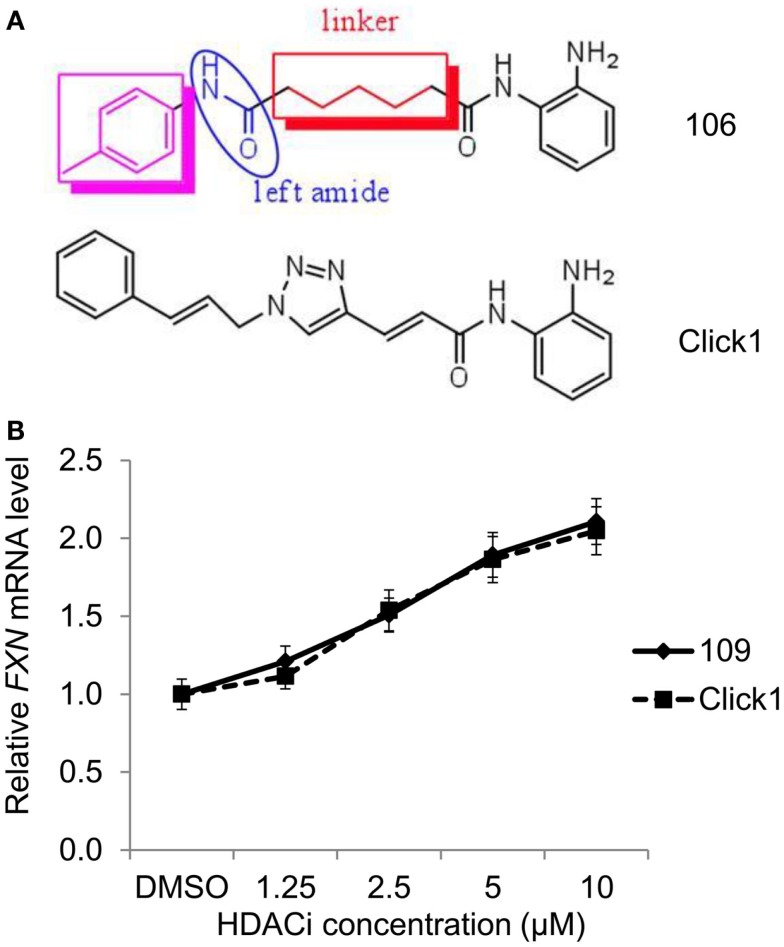
**HDAC inhibitors with improved pharmacological properties yield comparable increases in *FXN* mRNA in FRDA neuronal cells as the first-generation inhibitors**. **(A)** Structures of compound *106* and *Click-1*, showing replacement of the left amide in *106* with an unsaturated bond, and introduction of a triazole in the linker region in *Click-1*. An unsaturated bond was also introduced adjacent to benzamide to prevent benzimidazole formation, as observed for *106* and other pimelic 2-aminobenzamides. **(B)** Quantitative RT-PCR was used to compare the effects varying concentrations of HDACi *109* and *Click-1* on *FXN* mRNA in FRDA neuronal cells. mRNA levels were arbitrarily set to 1.0 for the DMSO control. Error bars = SEM of triplicate measurements.

Our first generation of molecules, such as *106*, *109*, and derivatives, suffer from two liabilities; namely, less than optimal brain penetration (0.15 brain to blood ratio) and conversion of the active molecule into an inactive metabolic product *in vivo* (a benzimidazole). To circumvent these liabilities, we identified two structural features that individually improve brain distribution and metabolic stability of our HDAC inhibitors. Brain penetration is improved by replacement of the “left” amide in the standard pimelic 2-aminobenzamide scaffold (Figure 1) with an ether, olefin (alkene), or ketone and introduction of a non-saturated α/β linkage adjacent to the “right” amide prevents formation of the benzimidazole. Based on these results, we used Cu(I)-catalyzed click chemistry to derive a small library of derivatives that combine these properties (31) (Figure 6A). To determine whether such molecules retain their ability to increase *FXN* mRNA in FRDA neuronal cells, we compared the activity of one such molecule (*Click-1*) to that of HDACi *109* (Figure 6B). *Click-1* contains two unsaturated linkages, one adjacent to the 2-aminobenzamide amide linkage and one replacing the “left” amide linkage in the original chemical scaffold. Notably, this molecule contains a triazole, which is the product of click chemistry. These changes to the HDACi scaffold were completely tolerated as evidenced by equivalent activities of HDACi *109* and *Click-1* in the transcription assay (Figure 6B). Given the improved pharmacology and specifically brain penetration of this new class of compounds (31), these findings offer promise for the development of a new generation of compounds for human clinical studies in FRDA.

## Discussion

We recently demonstrated (28) that HDACi *109* is able to reverse *FXN* gene silencing in FRDA neurons derived from patient iPSCs to a degree comparable to that found in earlier studies employing human PBMCs and mouse models (5, 23, 26, 27). Brain penetration and HDAC inhibition in the brain were established *in vivo* in two mouse models for FRDA (23, 27). Our recent study provided evidence for reversal of the heterochromatin state and up-regulation of *FXN* mRNA and frataxin protein in human neuronal cells. We also demonstrated HDAC inhibition and increased H3K9 acetylation in PBMCs and an increase in *FXN* mRNA in blood from patients treated with RG2833 (the drug formulation product of *109*). We demonstrated that combined HDAC1, 2, and 3 inhibition is required to counteract the epigenetic changes induced by the GAA•TTC repeat expansion and that H3K9 is a key histone residue whose acetylation/methylation regulates *FXN* expression. Compounds that are selective for particular class I HDACs do not reactivate *FXN* gene expression to the extent found for HDACi *109* and also do not promote H3K9 acetylation to a similar degree.

In the current study, we extend our previous observation that 4- and 5-substituted 2-aminobenzamides that are selective for HDAC3 or HDAC1, respectively, are relatively inactive as inducers of *FXN* gene expression in FRDA neuronal cells (28). We find that both HDACi *3* (this study) and *233* (28), which both target HDAC1, are weakly active compared to HDACi *109* (28). Similarly, the HDAC3-selective compounds *136* (this study) and *966* (28) are also inactive. HDACi *109* inhibits both HDAC1 and HDAC3 through a slow-on/tight-binding mechanism (mechanism 2 above), whereas the inactive 4- or 5-substituted 2-aminobenzamides do not share this property. For example, HDACi *136* exhibits slow-on/tight-binding for HDAC3, but is a fast-on/fast-off competitive inhibitor of HDAC1. Similarly, HDACi *3* only shows slow-on/tight-binding for HDAC1. Highly potent hydroxamate HDAC inhibitors such as SAHA and TSA are rapid-on/rapid-off inhibitors and do not reactivate *FXN* expression *in vitro* (5, 22). Based on our results with small-molecule inhibitors, we postulate that multiple HDACs may be responsible for *FXN* gene repression in FRDA, and that prolonged target engagement may be required for reactivation. Interestingly, combinations of HDAC1/2- and HDAC3-selective inhibitors do not reactive *FXN* gene expression (28), suggesting that reversal of epigenetic silencing can only be achieved by inhibition of the particular HDAC complexes residing at the *FXN* locus in FRDA cells.

To corroborate our findings with small-molecule HDAC inhibitors, we turned to siRNA silencing of class I HDACs. Strikingly, we find that siRNAs to either HDAC1 or HDAC3 yield comparable levels of *FXN* gene expression in FRDA neurons as observed with HDACi *109* (Figure 3). This finding suggests to us that the GAA•TTC repeat expansion may recruit a repressor complex containing both enzymes. The interplay between HDACs 1 and 3 in neurodegenerative diseases is well established as well as the interaction between HDAC1 and HDAC3 in neuronal cells [see for example Ref. (43)]. This suggestion would be in line with the small-molecule inhibitor findings.

It is also possible that our HDAC inhibitors act indirectly on the *FXN* gene by either up-regulating or down-regulating genes encoding chromatin modifying enzymes or proteins involved in chromatin structure. To assess this possibility, we analyzed microarray data from FRDA neuronal cells treated with HDACi *109*, *233*, and *966*. While approximately 1,200 genes are changed in expression by treatment of these cells with HDACi *109*, only 88 genes are uniquely regulated by *109*, and not by *233* and *966*. Among these, 12 genes were found to be involved in DNA binding, chromatin structure, or gene expression. Inspection of this list of genes (Table 3) revealed that two genes might be candidates for regulators of *FXN* gene expression, namely H2AFY2 and PCGF2. Since the histone variant macroH2A, encoded by the *H2AFY2* gene, is known to be involved in gene repression (41), down-regulation of this gene might well lead to increases in global gene expression, as well as increases in *FXN* gene activity. Interestingly, a recent study found that H2AY is specifically up-regulated in Huntington’s disease (HD) postmortem brain and lymphoid cells, and the levels of this mRNA are normalized by treatment of HD patients in human clinical studies with the HDAC inhibitors sodium butyrate and sodium phenylbutyrate (44). We find that our 2-aminobenzamide HDACi are also effective in reversing the global gene expression deficits in HD mouse models (29, 45). These findings may underpin a common mechanism of action of these compounds in FRDA and HD.

Similar to macroH2A, Polycomb group ring finger 2 is involved in transcriptional repression, being part of the Polycomb repressive complex 1 (PRC1). PRC1 and Polycomb repressive complex 2 (PRC2) are recruited sequentially to target genes to repress their expression (42). The components of PRC2, when recruited to target genes are responsible for the methylation of histone H3K27, providing a binding site for the chromobox domain of components of PRC1, which in turn catalyzes the monoubiquitination of H2AK119. H3K27 trimethylation is enriched at the *FXN* gene promoter and upstream and downstream of the GAA•TTC repeats in FRDA neuronal cells (28), suggesting that Polycomb-mediated repression may be involved in *FXN* gene silencing in FRDA. Hence, down-regulation of the Polycomb silencing complex could well lead to increases in *FXN* gene expression. Interestingly, a genome-wide association study of macroH2A in pluripotent cells revealed that its binding sites largely overlap with PRC2 binding sites (46). Additionally, previous studies have documented down-regulation of components of PRC2 with other HDAC inhibitors (47, 48). Future ChIP and siRNA experiments will be needed to assess occupancy of these gene products on *FXN* chromatin and the effects of the inhibitors on such occupancy.

While our present and recent findings (28) provide a proof of concept that patient-derived neuronal cells can be a quantitative screening tool for the development of an epigenetic therapy for FRDA, the compound used in our clinical study, *109*/RG2833, suffers from liabilities for chronic use as FRDA therapeutics; namely, less than optimal brain penetration (0.15 brain to blood ratio), and conversion of the active molecule into inactive, potentially toxic metabolic products (benzimidazole and amidolysis) that are poorly eliminated *in vivo* (31, 49). Other preclinical studies placed RG2833 and the benzimidazole in the high-risk category for inducing QTc prolongation (28). Given the cardiac involvement in FRDA, molecules that produce this benzimidazole metabolite are unlikely to be useful for chronic treatment of FRDA. We therefore searched for chemical modifications to the pimelic 2-aminobenzamide scaffold that would circumvent these liabilities. As described earlier (31), replacement of the “left” amide in the original scaffold (Figure 6A) with an ether, olefin, or double bond was found to improve brain penetration. Similarly, introducing a double bond adjacent to the benzamide prevented cyclization to the benzimidazole. These chemical features were combined into a small collection of derivatives by click chemistry (31), and compound *Click-1* was identified with improved brain penetration (>0.7 brain/plasma ratio) and stability to benzimidazole formation. We now provide evidence that *Click-1* retains its ability to activate *FXN* gene expression in FRDA neuronal cells (Figure 6B), with no loss of activity compared to HDACi *109*. Thus, new derivatives such as *Click-1* and other variants are candidates for future clinical studies in FRDA.

## Conflict of Interest Statement

James R. Rusche is an employee of Repligen Corporation. Joel M. Gottesfeld and C. James Chou are inventors on US patent applications 20070219244, 20110021562, 20130210918, and US patent 8,835,502 B2 (licensees, Repligen Corporation/BioMarin Pharmaceuticals). Joel M. Gottesfeld currently serves as a consultant to BioMarin Pharmaceuticals. The terms of this arrangement are managed by The Scripps Research Institute. Intellectual property has been licensed by The Scripps Research Institute to Repligen Corporation and BioMarin Pharmaceuticals, and The Scripps Research Institute, C. James Chou and Joel M. Gottesfeld have a financial interest in this technology.

## Supplementary Material

The Supplementary Material for this article can be found online at http://www.frontiersin.org/Journal/10.3389/fneur.2015.00044/abstract
