# Supplementary material for: Mechanism of Action of 2-Aminobenzamide HDAC Inhibitors in Reversing Gene Silencing in Friedreich’s Ataxia
Source: Front Neurol. 2015 Mar 5;6:44. doi: 10.3389/fneur.2015.00044 (PMC4350406; doi:10.3389/fneur.2015.00044)
Supplement: Supplementary file 1 [file Presentation_1.ZIP › presentation 1/Supplementary Figures 1-3.PDF]

Supplementary Figures 1 - 3. Inhibition mechanism for HDACi **109** (Supp. Fig. 1), HDACi **136** (Supp. Fig. 2) and HDACi **3** (Supp. Fig. 3) with HDAC1 (panel A) and HDAC3-NcoR (panel B). Plots of k_{obs} versus inhibitor concentration were derived from deacetylation assays, performed as described [22], at the indicated inhibitor concentrations. K_{obs} was obtained from fits to equation 1 of Chou et al. [22].

HDACi 109/HDAC1

A

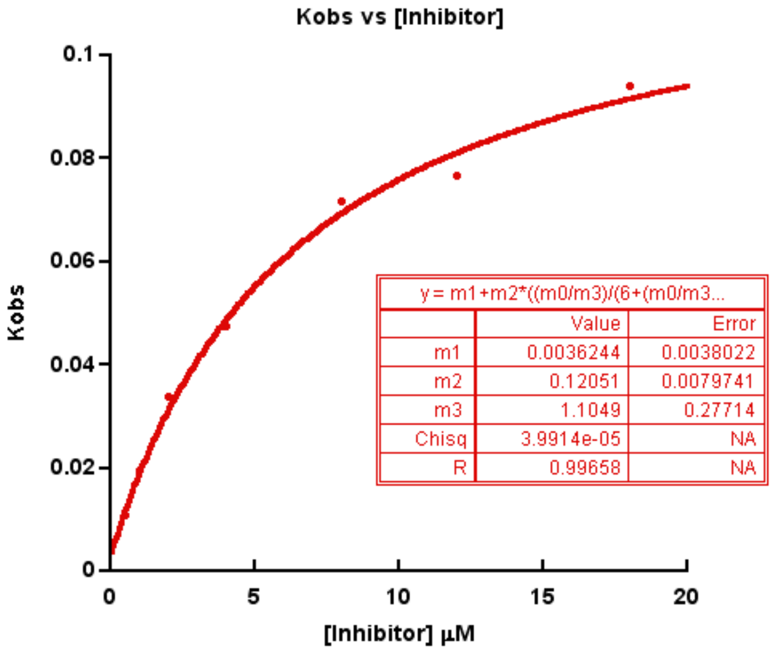

HDACi 109/HDAC3-NcoR

B

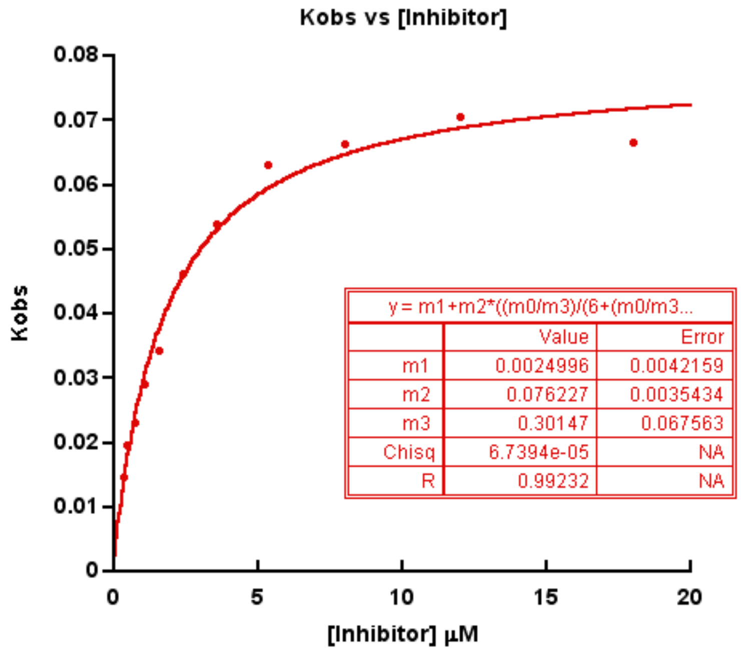

Supplementary Figure 1

HDACi 136/HDAC1

HDACi 136/HDAC3-NcoR

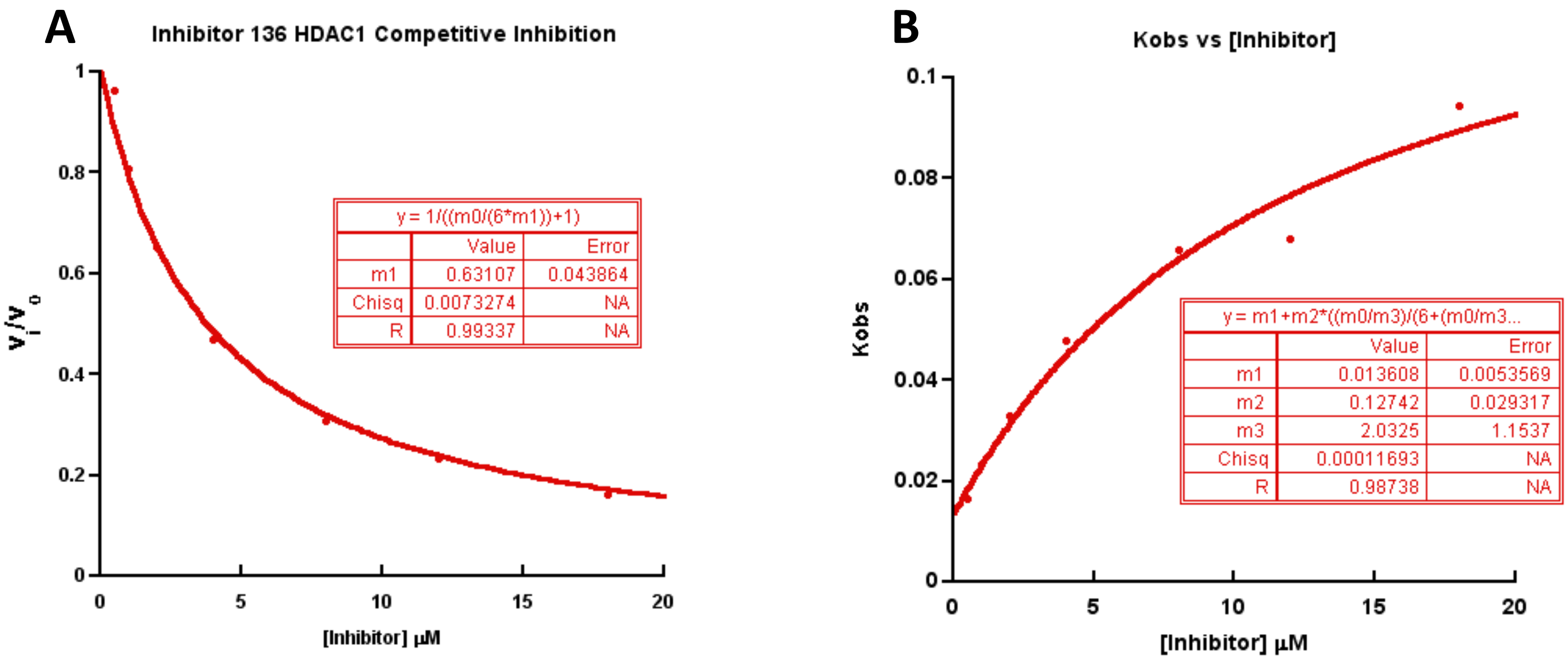

Supplementary Figure 2

HDACi 3/HDAC1

A

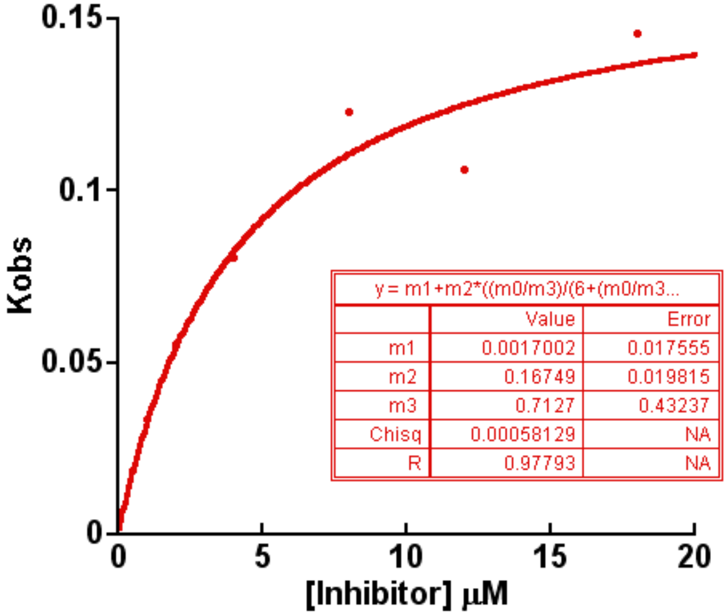

HDACi 3/HDAC3-NcoR

B

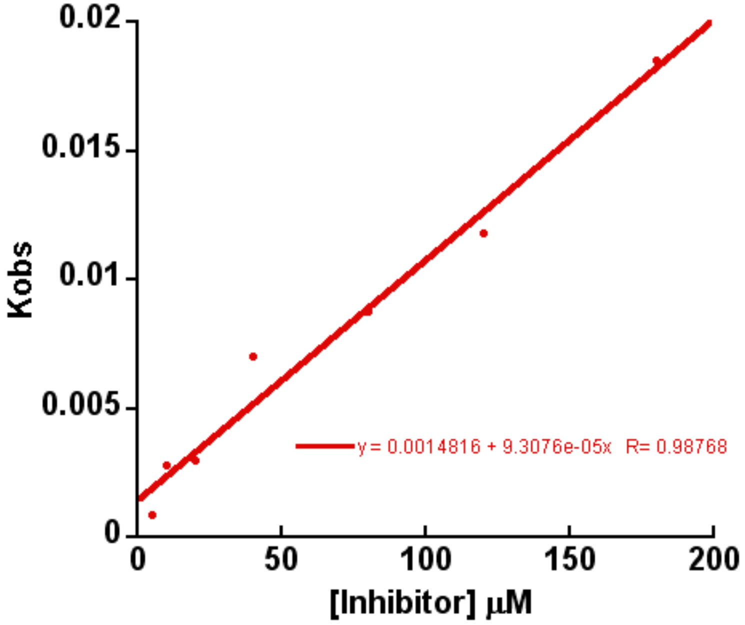

Supplementary Figure 3
